# The *carP* lncRNA Is a *carS*-Related Regulatory Element with Broad Effects on the *Fusarium fujikuroi* Transcriptome

**DOI:** 10.3390/ncrna7030046

**Published:** 2021-08-02

**Authors:** Javier Pardo-Medina, Gabriel Gutiérrez, M. Carmen Limón, Javier Avalos

**Affiliations:** Departamento de Genética, Facultad de Biología, Universidad de Sevilla, 41012 Seville, Spain; jpardo6@us.es (J.P.-M.); ggpozo@us.es (G.G.); carmenlimon@us.es (M.C.L.)

**Keywords:** RNA-seq, *carP* lncRNA, cis-acting mechanism, carotenoid biosynthesis, light regulation

## Abstract

Carotenoid biosynthesis in the fungus *Fusarium fujikuroi* is regulated by environmental factors, with light being the main stimulating signal. The CarS RING-finger protein plays an important role in the downregulation of structural genes of the carotenoid pathway. A recent transcriptomic analysis on the effect of *carS* mutation identified a gene for a long non-coding RNA (lncRNA) upstream of *carS*, called *carP*, the deletion of which results in increased *carS* mRNA levels and lack of carotenoid production. We have investigated the function of *carP* by studying the transcriptomic effect of its deletion and the phenotypes resulting from the reintroduction of *carP* to a deletion strain. The RNA-seq data showed that the loss of *carP* affected the mRNA levels of hundreds of genes, especially after illumination. Many of these changes appeared to be cascade effects as a result of changes in *carS* expression, as suggested by the comparison with differentially expressed genes in a *carS* mutant. Carotenoid production only recovered when *carP* was integrated upstream of *carS*, but not at other genomic locations, indicating a *cis*-acting mechanism on *carS*. However, some genes hardly affected by CarS were strongly upregulated in the *carP* mutant, indicating that *carP* may have other regulatory functions as an independent regulatory element.

## 1. Introduction

The genus *Fusarium* encompasses a large group of fungal species widely distributed in all ecosystems, most of them phytopathogens [1,2]. Some of them have been extensively investigated for the damage they cause in agriculture, but they are also compelling research models due to the richness of their secondary metabolism [3]. A prominent example is the rice pathogen *Fusarium fujikuroi*, well-known for its ability to produce gibberellins [4], plant growth-promoting hormones with applications in agriculture. This species can produce many other secondary metabolites, as indicated by the abundance of gene clusters in its genome dedicated to synthesizing a great diversity of compounds [5].

Recently, *F. fujikuroi* has received special attention as a model for the study of fungal carotenoid synthesis and its regulation [6]. All the genes involved in the synthesis of its main carotenoid, the carboxylic xanthophyll neurosporaxanthin, have been identified: *carB*, *carRA*, *carT,* and *carD*. Two of them, *carRA* and *carB*, are organized in a cluster (*car* cluster) together with *carX*, which encodes an enzyme that cleaves β-carotene to form retinal, and *carO*, which encodes a rhodopsin that uses retinal as a chromophore. Carotenogenesis in *Fusarium* is stimulated by light through transcriptional induction of the *carRA, carB,* and *carT* genes, and to a lesser extent, *carD* gene. Carotenoid photoinduction is the best known photoresponse in this fungus, but the details of its molecular mechanism remain largely unknown. The transcriptional induction of *car* genes by light is mediated by the White-collar protein WcoA. Inactivation of the *wcoA* gene severely impairs the transcription of these genes and, subsequently, the accumulation of carotenoids. However, carotenoid photoinduction is not completely abolished in the *wcoA* mutant. The phenotypic characterization of deletion mutants has revealed the accessory participation of two other photoreceptors, the DASH cryptochrome CryD and the small flavoprotein VvdA [7]. CryD appears to be responsible for a slower response through an unknown post-transcriptional mechanism, while VvdA regulates the activity of WcoA and possibly CryD as well. *Fusarium* genomes contain genes for other photoreceptors [8], including two rhodopsins, a phytochrome, and an additional cryptochrome. Except for CarO rhodopsin, involved in spore germination [9] and pathogenesis [10], the functions of these photoreceptors in *Fusarium* remain to be investigated.

A distinctive class of *F. fujikuroi* carotenoid mutants, generically called *carS,* exhibit intense orange pigmentation under all culture conditions due to the light-independent accumulation of large amounts of neurosporaxanthin and carotenoid precursors [11]. The *carS* mutants analyzed are affected in the *carS* gene (*FFUJ_08714*), which encodes a protein of the RING-finger family [12]. Their increase in carotenoid content is caused by the presence of larger amounts of transcripts of the structural genes of the carotenogenic pathway, particularly *carRA* and *carB*. The CarS protein is therefore a negative regulator of *Fusarium* carotenogenesis, although the *carS* mutation also affects the expression of genes of other cellular processes [13]. The mechanism of action of CarS is still unknown, but it has sequence similarity to CrgA from *Mucor circinelloides*. The deletion of *crgA* results in a carotenoid overproduction phenotype like that of the *carS* mutants in *F. fujikuroi.* CgrA exerts its function by modifying target proteins by ubiquitylation [14], suggesting a similar molecular mechanism for CarS.

Contrary to what might be expected from its role as a negative regulator of carotenogenesis, *carS* mRNA levels increase under illumination, although the induction is less pronounced than that of the structural *car* genes [12]. The regulation of *carS* may be of special relevance in the control of carotenogenesis in *F. fujikuroi*. The long upstream sequence of the *carS* gene has no predicted ORFs in the genome annotation; however, an RNA-seq study revealed an unidentified 1-kb transcript in this genomic region with features consistent with a long non-coding RNA (lncRNA). As implied by the RNA-seq procedure, this RNA, which we call *carP*, is presumably transcribed by RNA polymerase II and polyadenylated, but lacks protein-coding functions [15]. *carP* is transcribed from the same strand as the *carS* gene and is required for efficient expression of the *car* cluster genes, as indicated by the albino phenotype and the strong reduction in mRNA levels of the structural *car* genes produced by deletion of *carP* (Δ*carP*). Furthermore, an identical phenotype was observed in the close relative *F. oxysporum* when its equivalent *carP* sequence was deleted.

The regulatory function of *carP* is likely associated with the modulation of *carS* expression, as indicated by increased levels of *carS* transcripts in Δ*carP* mutants [15], which could explain the lower expression of structural *car* genes. However, the molecular mechanism by which *carP* performs its function is unknown. It is also unknown whether *carP* is involved only in the control of carotenogenesis or has other regulatory functions. To test this, we have investigated the consequences of *carP* deletion in the transcriptome of *F. fujikuroi*. We show that *carP* affects the expression of many other genes in addition to those of carotenogenesis. Furthermore, we have reintroduced the *carP* gene into a Δ*carP* mutant to check the influence of its genomic location on the restoration of the wild-type phenotype. The results showed that the effect of *carP* on carotenogenesis is *locus* dependent and consistent with a *cis*-acting role on *carS* regulation.

## 2. Results

### 2.1. RNA-seq Analysis of the Effect of ΔcarP Mutation

To analyze the possible regulatory functions of *carP* in addition to its presumed effect on the gene *carS* and the expression of structural carotenoid genes, total RNA was obtained from mycelia of the wild-type strain and the Δ*carP* mutant grown in the dark or illuminated for 60 min. This time of exposure to light was previously described as having a great impact on the transcriptome of this fungus [13]. It should be noted, however, that these conditions are far from the actual conditions in which the fungus thrives in nature, but they have been used in previous studies to investigate transcriptional light regulation. The RNA samples were subjected to massive sequencing and the relative amounts of mRNA were determined for all the annotated genes. The sets of transcripts were compared in the different relevant combinations, and the numbers of differentially expressed genes were determined using a threshold of 2x fold change (log2 = 1) (Table 1). Complete lists of genes exhibiting at least a 2x fold change of mRNA levels in all combinations are shown in Supplementary Table S1.

The data revealed a total of 1830 light-regulated genes in the wild-type strain, using the aforementioned threshold change in transcript levels between the illuminated and non-illuminated samples. The number of genes influenced by light was appreciably shortened in the Δ*carP* mutant, with 3.5-fold and 4.5-fold reductions for upregulated and down-regulated genes, respectively. In other words, about 75% of the transcripts that were upregulated by light in the wild-type strain, and about 81% of those that were downregulated, did not exhibit significant photoregulation in the Δ*carP* mutant. Comparison of transcript levels between the Δ*carP* mutant and wild-type strain showed a greater number of differentially expressed genes after illumination than in the dark, with a greater abundance of downregulated genes in the mutant.

**Figure 1:**
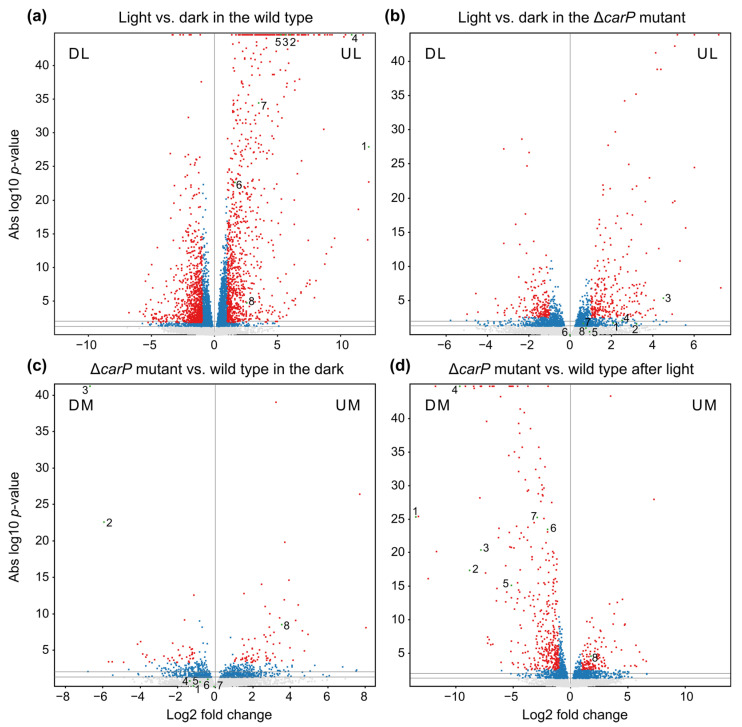
Volcano plot representations of global expression data in the indicated pairwise comparisons. (**a**,**b**) Effect of 1 h illumination in the wild type (**a**) and in the Δ*carP* mutant (**b**). DL: downregulated by light; UL: upregulated by light. (**c**,**d**) Effect of Δ*carP* mutation in the dark (**c**) or after 1 h illumination (**d**). DM: downregulated in the Δ*carP* mutant; UM: upregulated in the Δ*carP* mutant. *p*-values of 0.05 and 0.01 are indicated with grey horizontal lines. Genes with a *p*-value < 0.05 are indicated in blue. Fold changes in abscissae are obtained from DESeq2 data. Genes exceeding the log2 values of ±1 are indicated in red. Genes related to carotenoid metabolism are marked with green dots and a number. 1: *carX* (*FFUJ_11801*); 2: *carRA* (*FFUJ_11802*); 3: *carB* (*FFUJ_11803*); 4: *carO* (*FFUJ_11804*); 5: *carT* (*FFUJ_07962*); 6: *carD* (*FFUJ_07503*); 7: *ggs1* (*FFUJ_07352*); 8: *carS* (*FFUJ_08714*).

Differences are more clearly seen in graphical representations, such as volcano plots (Figure 1) or scatter plots (Supplementary Figure S1). Even though similar numbers of genes were upregulated and downregulated by light, the greatest changes in mRNA levels were observed mainly among the photoinducible genes. However, the larger effects disappeared in the Δ*carP* mutant (compare Figure 1a,b and Supplementary Figure S1a,b), especially for photoinduced genes. On the other hand, consistent with the data displayed in Table 1, comparisons between sets of wild-type strain and Δ*carP* mutant transcripts under the same conditions showed a higher impact of the *carP* mutation after illumination than in the dark (Figure 1c,d and Supplementary Figure S1c,d).

Due to the effect of the *carP* deletion on the regulation of carotenoid synthesis, the points corresponding to the four genes of the *car* cluster: *carRA*, *carB*, *caX*, and *carO*, as well as those for the early gene *ggs1*, the late genes of the synthesis of neurosporaxanthin, *carT* and *carD*, and the regulatory gene *carS*, have been highlighted in Figure 1 and Supplementary Figure S1. In accordance with the former results [6], the Δ*carP* mutation not only decreases the response to light of the structural *car* genes, which is also patent in the comparisons between wild-type strain and Δ*carP* mutant under light, but also that of *ggs1*. This gene encodes geranylgeranyl diphosphate synthase, which performs the reaction prior to the synthesis of phytoene, the first carotene in the pathway (see Section 2.6). The *carS* gene, unlike the other genes already indicated, increases its mRNA levels in the Δ*carP* mutant both in the dark and after illumination, which confirms our previous observations [15].

### 2.2. Genes Affected by Light in the Wild-Type Strain and in the ΔcarP Mutant

**Figure 2:**
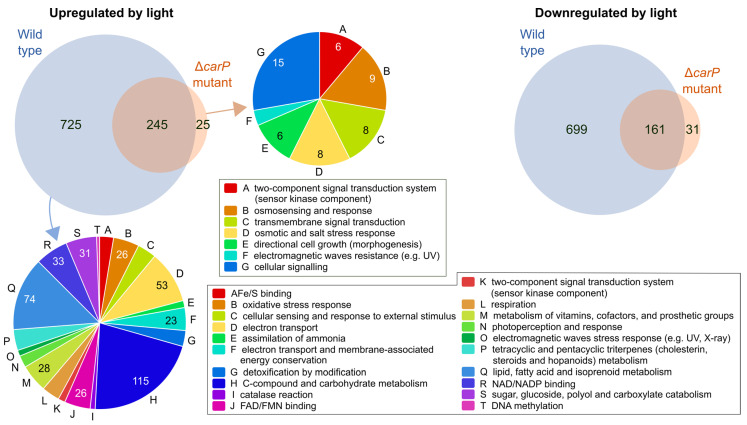
Effect of light in the transcriptomes of the wild type and the Δ*carP* mutant. Venn diagrams show the proportion of coincident genes between those upregulated (**left**) or downregulated (**right**) by light in both transcriptomes. Funcat categories are shown for the three transcript sets for which significant categories were found.

Comparison of the lists of light-affected genes in the two strains showed that about 90% of the genes photoinduced in the Δ*carP* mutant were also photoinduced in the wild-type strain, a proportion that was also very high (*ca*. 83%) for photorepressed genes (Figure 2). As expected from previous analyses under similar conditions [13], genes induced by light in the wild-type strain were involved in very different physiological processes, including some related to stress. However, the functional categories that were found relevant in genes that exhibited photoinduction in the Δ*carP* mutant were enriched for activities related to signaling pathways. Functional assignations were less clear among the photorepressed genes in the wild-type strain, some of them related to cell differentiation, virulence, and secondary metabolism (Supplementary Figure S2), and no significant functional groups were identified among the photorepressed genes in the Δ*carP* mutant.

### 2.3. Genes Differentially Expressed in the ΔcarP Mutant Compared to the Wild-Type Strain

**Figure 3:**
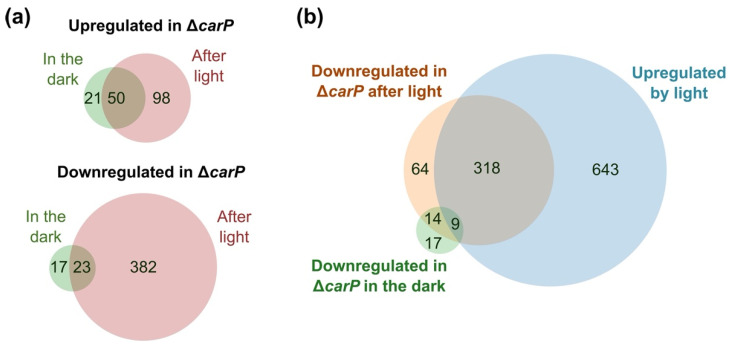
Effect of the Δ*carP* mutation in the dark or after one hour illumination. (**a**) Venn diagrams show the proportion of coincident genes between those upregulated (above) or downregulated (below) in the Δ*carP* mutant in the dark or after one hour of light. (**b**) Venn diagram showing the coincidences between genes upregulated by light in the wild-type strain and those downregulated in the Δ*carP* mutant.

As mentioned above, illumination caused an increase in the number of differentially expressed genes in the Δ*carP* mutant compared to the wild-type strain. More than half of the genes upregulated or downregulated in the mutant in darkness were also upregulated or downregulated after illumination (Figure 3a). Analysis of the enriched GO categories found those for carotenoid metabolism among the downregulated ones, but only in the dark because of differences in statical relevance due to the large differences in gene numbers (Supplementary Figure S3). Interestingly, a high proportion of the genes downregulated in the Δ*carP* mutant under light were upregulated by light in the wild-type strain under the same conditions (Figure 3b), indicating that *carP* is needed for adequate photoinduction of a large collection of genes.

**Figure 4:**
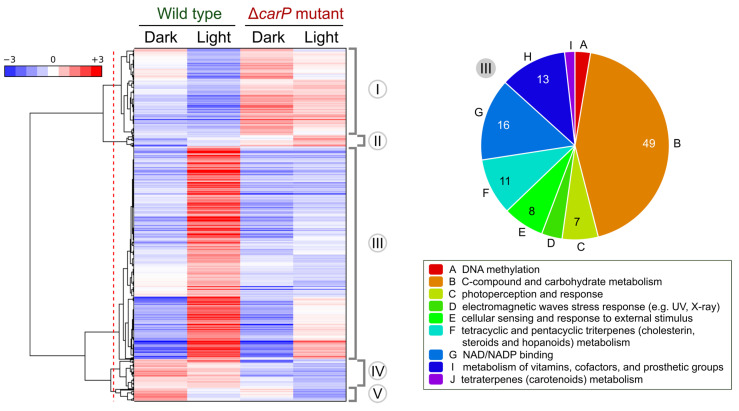
Hierarchical heatmap for genes influenced by *carP* in the transcriptomes of the wild type and the Δ*carP* mutant. The red line indicates an arbitrary threshold distinguishing five cluster groups, from I to V, indicated on the right. Significant FUNCAT categories found in cluster III are shown on the right.

To obtain information on the possible functions of the genes influenced by *carP*, we carried out a hierarchical heatmap according to the mRNA levels of the 591 genes that exhibited a change with respect to the wild-type strain in at least one of the two conditions tested (Figure 4). The tree is separated into two main branches, which correspond to genes that were upregulated or downregulated in the Δ*carP* mutant relative to the wild-type strain. For a more specific analysis, a discrimination threshold was arbitrarily chosen (red dotted line in the figure) to distinguish five clusters, two corresponding to upregulated genes (I-II) and three to downregulated genes (III-V). The lists of genes in each cluster are described in Supplementary Table S2.

Cluster I consists of 145 genes that, in most cases, are downregulated by light in the wild-type strain. Although their expression patterns were quite diverse, they all showed higher transcript levels in the Δ*carP* mutant either in the dark or after illumination, and in many cases photorepression was preserved. GO enrichment analysis only found as significant 26 genes belonging to the category “C-compound and carbohydrate metabolism” (Supplementary Table S3). On the other hand, Cluster II includes only 19 genes with a predominance of photoinduction in the wild-type strain, which, like those of cluster I, are upregulated in the Δ*carP* mutant. Six of the genes were found to be involved in “sugar, glucoside, polyol, and carboxylate anabolism”. Therefore, all genes in clusters I and II with significant associations with GO categories were related to metabolism.

Cluster III is the major class, covering 357 genes induced by light in the wild-type strain and downregulated in the Δ*carP* mutant. This group included genes associated with nine GO categories (Figure 4), corresponding to very diverse functions. The genes for carotenoid biosynthesis, which are known to be repressed in the Δ*carP* mutant [15], and other genes regulated by CarS (see Section 2.6), belong to this category.

Clusters IV and V include genes that were downregulated in the Δ*carP* mutant, but in this case, they were only moderately affected by light (cluster IV) or clearly downregulated (cluster V) in the wild-type strain. No significant GO categories were found in the 49 genes of cluster IV, which included the genes for the nitrate reductase NiaD (*FFUJ_12277*, repressed about 10-fold in the Δ*carP* mutant), and the probable nitrate transport protein CrnA (*FFUJ_00934*, repressed about 5-fold). On the other hand, the 20 genes of cluster V included 4 genes associated with the GO category “polysaccharide metabolism”.

### 2.4. Reintegration of carP in a ΔcarP Mutant

In a recent work, the albino phenotype of the Δ*carP* mutant was described [15]. To confirm that this trait is due to a lack of *carP*, we reintroduced the wild-type *carP* gene into a Δ*carP* mutant. In *F. fujikuroi* transformation experiments, plasmids containing homologous sequences are typically integrated into the genome by homologous recombination at the native site or by heterologous recombination at random locations in the genome. Since the mechanism of action of *carP* is unknown, analysis of both types of transformants could provide valuable information on whether *carP* RNA acts in *cis* or in *trans*. Therefore, we constructed a transformation vector, pRS246carPneo, which contains the native *carP* sequence (including its presumptive promoter and terminator sequences) and a G418 resistance cassette (*neoR*). pRS246carPneo was sequenced to ensure the integrity of the *carP* sequence, and only a change was detected, the loss of a cytidine close to a C-rich segment, at −166 bp upstream from the *carP* transcription initiation (marked in Supplementary Figure S4), which was not expected to affect *carP* function.

**Figure 5:**
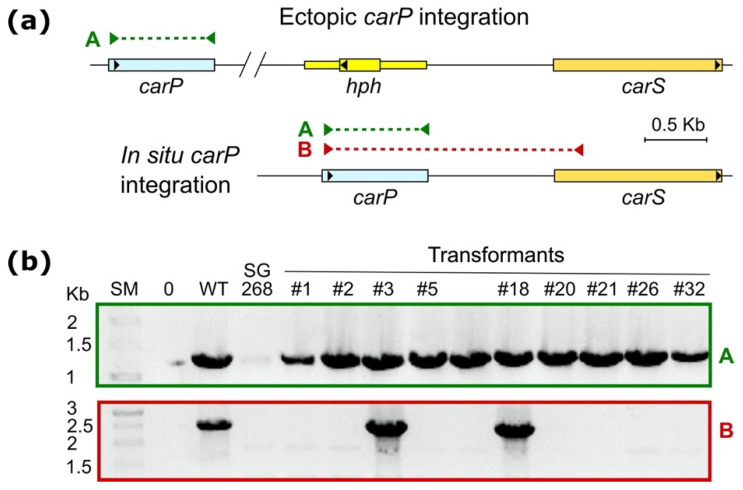
PCR analysis of the reintegration of the *carP* sequence in SG268. (**a**) Genomic maps of ectopic and in situ *carP* integrations in the SG268 Δ*carP* mutant. Yellow (*hph*): hygromycin B resistance cassette that replaced the *carP* gene in SG268 [15]. (**b**) Gel electrophoresis of PCR products from candidate transformants to check the insertion of the *carP* sequence. Wild-type strain DNA (WT) was used as positive control, and SG268 (Δ*carP* mutant) or lack of DNA (0) were used as negative controls. PCR amplification products are indicated on the top map with colored arrowheads and dotted lines. The expected PCR sizes are 1245 bp (A, green), and 2194 bp (B, red).

Protoplasts of the Δ*carP* mutant SG268 were incubated with linearized pRS246carPneo plasmid and G418-resistant colonies were selected, resulting in the isolation of 32 presumptive transformants. Ten of them were genetically purified by growth of uninucleate microconidia in three successive steps and analyzed by PCR with different primer combinations to check the presence of the *carP* gene (Figure 5a and Supplementary Figure S4a). All G418 resistant transformants were confirmed to contain the *carP* sequence. However, the integration occurred in the native *carP* locus in only two of them, T3 and T18, as indicated by the expected PCR amplification between the *carP* and *carS* sequences from their genomic DNA samples (Figure 5b and Supplementary Figure S4b). This result was confirmed in a Southern-blot hybridization, in which the bands corresponding to the integration of the plasmid by homologous integration upstream of *carS* were only found in T3 and T18, but not in other representative transformants (Supplementary Figure S4c). The combined data from Southern-blot hybridization and PCR amplification with the appropriate probe and primers strongly support integration of *carP* upstream to *carS* at the same distance as in the wild-type strain, in T3, and in T18. However, the Southern blot also showed other hybridizing bands in T3 and T18, indicating additional ectopic plasmid integrations. Transformants T3 and T18, along with T2 and T21, used as controls for lack of *carS*-linked *carP* integrations, were used to perform detailed phenotypic analyses.

### 2.5. Phenotypic Characterization of Transformants Expressing carP in a ΔcarP Mutant

**Figure 6:**
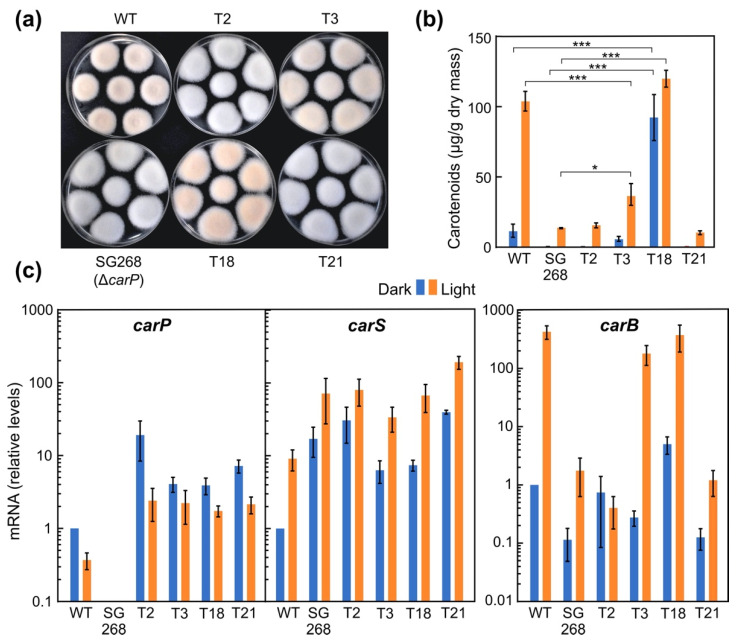
Effects of *carP* reintegration in the Δ*carP* mutant SG268. (**a**) Aspect of the colonies of the wild-type strain (WT), the Δ*carP* mutant SG268, and the transformants T2, T3, T18, and T21, grown for 1 week under light. (**b**) Carotenoid content in the same strains grown for 1 week in the dark or under light. Differences found to be significant according to the *t* tests are indicated (*p*-values, *: *p* < 0.033; ***: *p* < 0.001). (**c**) Transcript levels for the *carP*, *carS*, and *carB* genes in the same strains grown in the dark (blue bars) or exposed to light for 1 h (orange bars). RT-qPCR data show the mean and standard error of three independent experiments. Transcript levels were normalized against those of the β_1_-tubulin *FFUJ_04397* and GPDH *FFUJ_13490* genes. Relative mRNA levels are referred to the mRNA content of the wild-type strain in darkness.

No differences in growth or morphology were noted between the colonies of the transformants and the parental strain. However, the T3 and T18 strains showed an orange pigmentation, clearly distinguishable from the albino phenotype exhibited by all other transformants, of which T2 and T21 are representative examples (Figure 6a). Carotenoid analyses confirmed the visual phenotype of the mycelium, with increased levels of carotenoids only in T3 and T18 compared to other transformants or the original SG268 Δ*carP* mutant (Figure 6b). However, there were differences in the carotenoid content between T3, T18, and the wild-type strain. While the carotenoid production by T3 resembled that of the wild-type strain in the dark, it only accumulated a third of its carotenoid content in the light. In contrast, T18 exhibited a different pattern of carotenoid accumulation: its levels were like those of the wild-type strain under light, but were much higher in the dark, similar to those by the same strain under illumination. Regardless of these differences, presumably due to side effects of *carP* expression at other integration sites or differences in *carP* transcription in the native locus, it is noteworthy that the ability to produce carotenoids was only recovered when *carP* was integrated into its own genomic location. The fact that *carP* must be upstream of *carS* to exert its regulatory role in carotenogenesis suggests that this lncRNA is a *cis*-acting regulatory element.

The mRNA levels of the structural *carB* gene, and the regulatory genes, *carS* and *carP*, were analyzed in the wild-type strain and the four transformants described above (Figure 6c). The *carB* mRNA levels matched the carotenoid content of the different strains. Thus, the *carB* transcript was found in lower amounts in the T2 and T21 transformants than in the wild-type strain, and the levels were like those found in the original SG268 mutant. However, in T3 and T18 the *carB* mRNA levels were as high after illumination as those of the wild-type strain, and in the case of T18, even higher in the dark, correlating with the high content of carotenoids under these conditions.

As expected from the integration of plasmid pRS246carPneo, *carP* expression was restored in all four transformants, regardless of the genomic location. However, all strains contained a higher content of *carP* RNA, consistent with the presence of more than one integrated copy, as indicated by the additional bands observed in the Southern blot (Supplementary Figure S4c). As observed in the wild-type strain, *carP* transcript levels were appreciably higher in the dark than after illumination in all transformants.

The mRNA levels of *carS* gene showed a 5- to 10-fold increase after illumination compared to dark controls in all strains tested, a result consistent with former observations for *carS* regulation [13]. Regardless of the effect of light, the amount of *carS* mRNA was markedly higher in all transformants, including the *carP* mutant SG268, compared to those of the wild-type strain. The lower *carS* mRNA levels seemed apparent in transformants T3 and T18 compared to T2, T21, and SG268, in the dark, although the amounts were still clearly above those of the wild-type strain. Considering the high carotenoid content of T18, the high mRNA levels of *carS* in T18 in the dark in relation to the wild-type strain suggest a post-transcriptional regulation that remains to be elucidated.

### 2.6. Relation of the Effects of carP and carS Mutations

**Figure 7:**
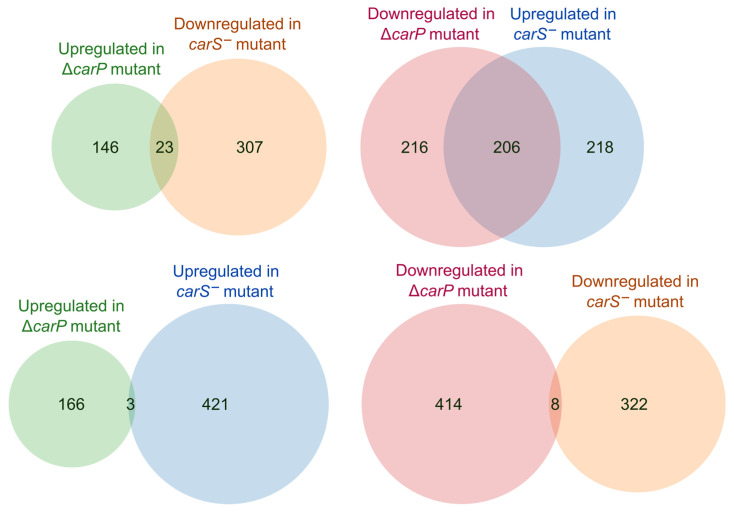
Relation between the genes affected by Δ*carP* or *carS^−^* mutations. Venn diagrams show the proportion of overlapping genes between those upregulated or downregulated in either combination between the tested mutants. Data for *carS^−^* mutant taken from RNA-seq analyses of the effect of the *carS* mutation in strain SG39 [13].

In the *carP* complementation experiments, the carotenoid-producing phenotype was only recovered when the wild-type *carP* gene was integrated at its own genomic location, suggesting that *carP* modulates *carS* expression via a *cis*-acting mechanism. A recent study of the effect of the *carS* mutation on the transcriptome showed that CarS influences the expression of a large set of genes [13]. This hypothesizes that the effects of *carP* deletion in the transcriptome could be due to its influence on *carS* expression. To test this hypothesis, we compared the sets of genes affected by both mutations using the same 2x differential expression threshold. The numbers of affected genes differed appreciably in both cases (upper Venn diagrams in Figure 7): loss of *carS* function resulted in the upregulation of 424 genes, compared to 170 in the case of the loss of *carP*, and the downregulation of 330, compared to 423 in the case of *carP*. Interestingly, the number of genes upregulated in the *carS* mutant (424) and downregulated in the *carP* mutant (423) was very similar, and about half of them coincided. However, the proportion of matches was much lower in the opposite combination (upregulated in Δ*carP* vs. downregulated in *carS^−^*). In contrast, the overlap between the affected genes in both strains was basically non-existent if the effects of the mutation were the same (bottom Venn diagrams in Figure 7).

**Figure 8:**
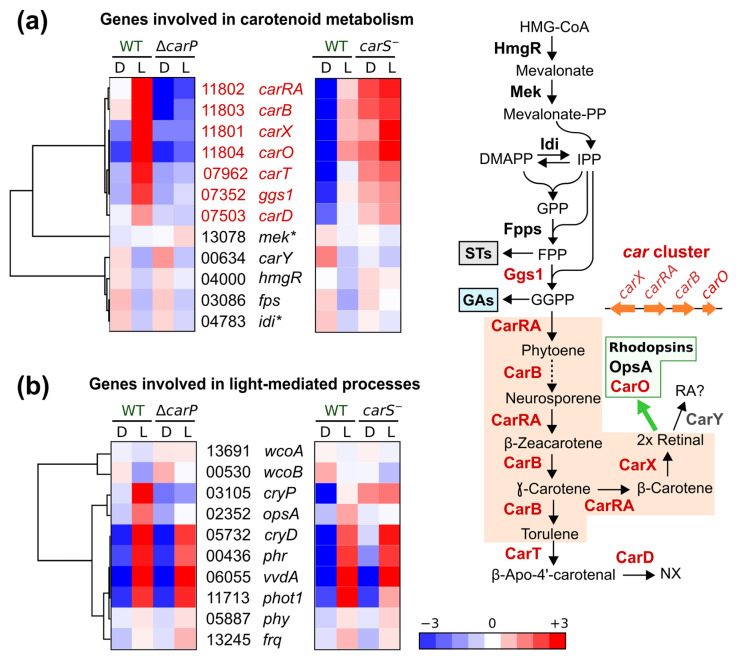
Relation between the effects of the Δ*carP* or the *carS^−^* mutations on the expression of the genes related with carotenoid metabolism or with regulation by light. (**a**) Hierarchical maps of the relative transcript levels for the genes involved in carotenoid metabolism. Reactions carried out by each enzyme are depicted on the right panel. Enzymes whose genes are regulated by light are indicated in red. The green box highlights the rhodopsins OpsA and CarO as presumed users of retinal as prosthetic group. The dotted lines indicate more than one reaction. HMG-CoA: hydroxymethyl glutaryl coenzyme A; DMAPP, dimethyl allyl diphosphate; IPP: isopentenyl diphosphate; GPP: geranyl diphosphate. FPP, farnesyl diphosphate; GGPP, geranylgeranyl diphosphate; STs, sterols; GAs, gibberellins; RA?, possible formation of retinoic acid by enzyme CarY [16]. For functions of enzymes for early steps in terpenoids biosynthesis, HmgR, Mek, Idi, Fpps, and Ggs1, see [6,17]. (**b**) Hierarchical maps of the relative transcript levels for the genes involved in light regulation. In (**a**,**b**) the numbers correspond to the *FFUJ*_ codes in the genome.

The *carS* gene product downregulates the expression of all genes involved in NX and retinal biosynthesis in *F. fujikuroi* (Figure 8a). All these genes, as well as the rhodopsin *carO* gene, are also upregulated by light (genes and enzymes indicated in red in the figure). Comparison of the effects of *carS* and *carP* mutations showed opposite effects in these genes, with a clear downregulation for all of them in the absence of *carP*. Such an effect was particularly strong in the case of the genes of the *car* cluster, which includes the *carO* gene. Only slight effects were appreciated for genes corresponding to the first steps of terpenoid biosynthesis, from HMG-CoA to FPP, shared with sterol synthesis. Interestingly, the *carY* gene, presumably involved in retinoic acid production from retinal [16], exhibited the opposite pattern, but the effect was less pronounced.

Due to the relation of *carP* to photoregulated genes, the effects of the *carS* and *carP* mutations on the mRNAs of genes encoding photoreceptors were also investigated and compared with equivalent data from RNA-seq analyses of the *carS* mutant (Figure 8b). Furthermore, the gene for the orthologous protein to Frq of *N. crassa*, involved in circadian rhythmicity, was also included in this analysis due to its connections with light regulation in other fungi [18]. The heatmaps showed that the genes encoding the DASH cryptochrome CryD, the photolyase Phr, the flavoprotein VvdA, and the recently identified putative phototropin Phot1 [17], were strongly induced by light. However, such inductions were not affected by *carP* lncRNA, and they were not essentially affected by CarS either. However, the gene for the cryptochrome CryP, and to a lesser extent also the gene for the rhodopsin OpsA, were downregulated in the *carP* mutant. The *carS* mutation produced the opposite effect in *cryP* mRNA levels, but not in those of *opsA*, for which a similar downregulation was also observed in the *carS* mutant. Therefore, the alteration in *cryP* mRNA levels in the Δ*carP* mutant could be a cascade effect of the role of *carP* in *carS* regulation. No clear patterns of change were found in the other genes investigated, those for the white-collar proteins WcoA and WcoB, the phytochrome Phy, and the putative clock protein Frq.

**Figure 9:**
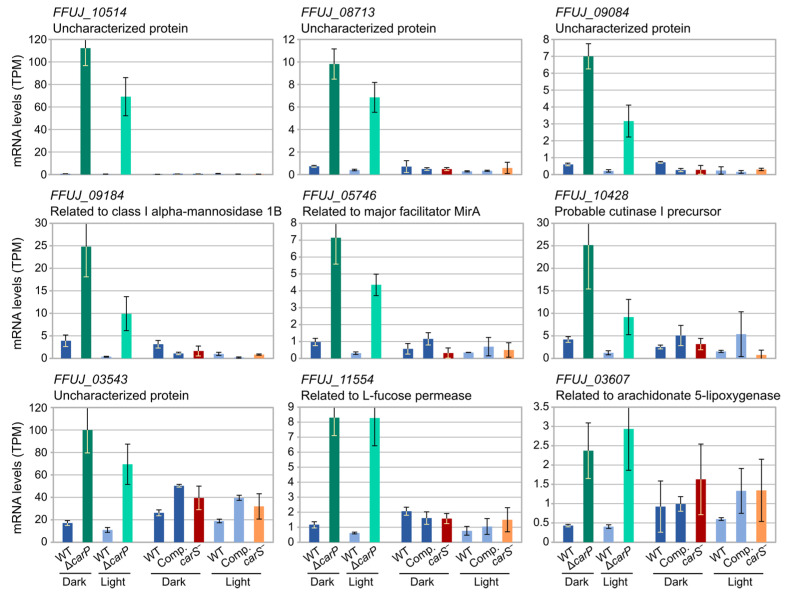
Examples of genes differentially expressed in the Δ*carP* mutant that were not affected by *carS* mutation. Transcriptomic data for the effect of the *carS* mutation were extracted from data sets already available [13]. WT: Wild-type strain IMI58289. *carS^−^*: *carS* mutant SG39. Comp: complementation of SG39 with wild-type *carS* allele. Light: 1 h illumination.

Interestingly, the comparison of our RNA-seq data with those previously available on the influence of the *carS* mutation in the transcriptome shows the existence of genes that were clearly affected by the *carP* deletion but in which the *carS* mutation had no important influence. Examples were mostly found in the case of genes upregulated in the Δ*carP* mutant (Figure 9). Among them, there were several genes of unknown function with a sharp increase in their mRNA levels in the absence of *carP*, either in the dark or after illumination, while there were no apparent changes in the *carS* mutants. Although with not so strong effects, a similar result was obtained for some photorepressed genes, such as those for a protein related to a class I-alpha-mannosidase 1B (*FFUJ_09184*), major facilitator MirA (*FFUJ_05746*), or a cutinase I precursor (*FFUJ_10428)*.

## 3. Discussion

In a previous study, the existence of the *carP* gene was demonstrated in *F. fujikuroi* [15], which determines a lncRNA that exerts its function as an independent transcript upstream of the *carS* gene, which encodes a carotenogenesis repressor protein. The loss of *carP* results in a lack of pigmentation due to a drastic reduction in the synthesis of carotenoids and the expression of their structural genes, indicating a regulatory role in this biosynthetic pathway. It has recently been shown that enhanced *carS* transcription obtained by expressing promoter-engineered versions of *carS* also leads to an albino phenotype [19]. This result, together with the overproducing phenotype resulting from loss of *carS* function [12], indicates that *carS* expression is finely tuned in *F. fujikuroi* to allow an adequate response of carotenoid synthesis in response to light. An obvious hypothesis for the albino phenotype of the Δ*carP* mutant is that it is due to enhanced levels of *carS* mRNA, and that in the wild-type strain *carP* exerts a negative regulatory action on the downstream *carS* gene.

The reintroduction of the *carP* sequence in the *ΔcarP* mutant has provided valuable information on its possible mechanism of action. Despite the varying behavior of the analyzed transformants, presumably due to differences in the expression of *carP* in the different genomic integrations, carotenoid production was only recovered in the transformants in which the *carP* gene was introduced in its native site. The rest of the transformants were albinos even though they were able to transcribe *carP*, strongly supporting *carP* as a *cis-*regulatory element that acts on *carS* expression. The results are consistent with the higher levels of *carS* mRNA observed in the Δ*carP* mutant and in the ectopic transformants T2 and T21, which resemble the *carS* overexpression obtained with the use of a stronger promoter [20]. The *cis*-acting function is also against an interference effect of *carP* on a transcription factor involved in *carS* expression, which could be an alternative explanation to the enhanced *carS* expression in the absence of *carP*.

Transcriptional interference is a well-known mechanism of action for lncRNAs and, in fact, it is the most common mechanism described for lncRNAs in *S. cerevisiae* [21]. Interference can be achieved through synthesis as a sense upstream transcript, as seems to be the case for *carP*, or as an antisense overlapping transcript. The first consists of the downregulation of a neighboring downstream gene due to the disturbance that lncRNA synthesis produces on the start of its transcription process. A well-known example in *S. cerevisiae* is *SRG1*, which in this way regulates the *SER3* gene, encoding an enzyme necessary for serine biosynthesis [22]. There is at least another *cis*-acting mechanism that allows the deregulation of a neighboring gene. *carP* could act as a scaffold to facilitate the assembly of histone-modifying enzymes and alter the expression of *carS*. An example of such a *cis*-acting element is the *GAL10* lncRNA in *S. cerevisiae* [23]. Under conditions of repression caused by glucose, the production of *GAL10* lncRNA stimulates the di- and trimethylation of K4 and the dimethylation of K36 on histone 3 by Set2. These are repressive chromatin marks that are bound by Eaf3, which recruits histone deacetylase Rpd3S, resulting in extensive deacetylation and silencing of the entire *GAL* locus.

Transcriptomic analysis of the effect of the Δ*carP* mutation revealed its influence on the expression of many genes other than those of carotenogenesis. This is not surprising, considering the broad regulatory effects of the *carS* mutation. Previous RNA-seq studies performed with a *carS* mutant demonstrated that CarS influences the expression of an extensive battery of genes [13]. Consistent with the regulatory role of *carP* on *carS*, cascade effects are expected in the Δ*carP* mutant reminding those produced by loss of *carS* function. Interestingly, the transcriptomic effects of the Δ*carP* mutation augmented after illumination. Data on the influence of CarS on the *F. fujikuroi* transcriptome showed regulatory connections between CarS and photoregulation, as indicated the high coincidence between genes influenced by this protein and those affected by light [13]. In addition, the number of light-regulated genes decreased significantly in the *carS* mutant, 3.5-fold for the photoinduced genes and 4-fold for the photorepressed genes. These reductions were like those observed in the Δ*carP* mutant, 3.6-fold and 4.5-fold, respectively, which again agrees with an effect of *carP* through *carS* expression. Photocarotenogenesis, like other photoresponses in this organism, is mediated by the WcoA protein [19], presumably in coordination with the WcoB protein, forming a complex. However, the loss of *carP* did not noticeably affect the levels of *wcoA* or *wcoB* mRNA, nor did it affect those of the genes of other photoreceptors that also participate in the regulation of carotenogenesis by light, such as CryD and VvdA [7]. In the case of the *cryD* gene, the lack of effect of the Δ*carP* mutation on its mRNA levels differed from the clear downregulation observed in the *carS* mutant [13].

Apart from the aforementioned similarities, there were notable differences between the influence of *carS* and *carP* mutations on the transcriptome: while the *carS* mutation caused large changes in differentially expressed genes compared to the wild-type strain in the dark (4.7% upregulated and 5.4% downregulated) or after illumination (3.3% and 4.2%, respectively), the numbers of upregulated and downregulated genes in the Δ*carP* mutant were much lower (0.4% and 0.2% in the dark, and 1.0 and 2.7% after illumination, respectively). In fact, RNA-seq analyses on the effect of the *carP* deletion showed a correlation with the effects of the *carS* mutation only for genes whose expression is decreased in the Δ*carP* strain, including those of carotenogenesis. However, much fewer matches were found between the genes affected by both mutations in the case of those with higher mRNA levels in the Δ*carP* mutant. Detailed analysis of the expression data showed the existence of genes strongly upregulated by *carP*, without significantly changing in the *carS* mutant, of which some examples have already been shown. Regardless of the action of *carP* as a regulatory element of *carS*, the existence of these specific effects of *carP* suggests that this lncRNA can exert repressive actions on other genes without the mediation of the CarS protein, most likely as a free RNA molecule. Genes that are affected by *carP* independently of *carS* may be the result of direct effects of *carP*, or secondary effects resulting from the control by *carP* of the expression or activity of other regulatory proteins. Thus, *carP* may act on other regulatory protein through a mechanism like that described for the *HAX1* lncRNA of *T. reesei* [24]. *HAX1* is a transactivator of cellulase expression that forms an RNA–protein complex with the regulatory protein Xyr1 [25].

In summary, the available information points to *carP* lncRNA as a *cis*-acting regulatory element of the *carS* gene, possibly through a transcriptional interference mechanism. However, the transcriptomic data also point to *carP* as an independent regulatory lncRNA unrelated to the control of *carS* expression. More studies are needed to understand the biochemical mechanism by which *carP* exerts its function as a free RNA and to identify its specific molecular targets.

## 4. Materials and Methods

### 4.1. Strains and Culture Conditions

The wild-type strain of *Fusarium fujikuroi* IMI58289 was obtained from the Imperial Mycological Institute (Kew, Surrey, England). The Δ*carP* mutant SG268 was obtained by replacing the transcribed *carP* region with a hygR resistance cassette [15]. The strains were cultured in DG minimal medium under the indicated conditions, except for conidia production, in which case the cultures were carried out on EG medium for 7 days [20]. For expression studies, the strains were grown in 100 mL of DG medium in 500 mL Erlenmeyer flasks, inoculated with 10^6^ conidia. The flasks were kept in total darkness for three days at 30 °C on an orbital shaker (150 rpm). The cultures were then transferred to four 25-mL Petri dishes under safe red light and kept in static conditions in the dark for 240 min. Subsequently, the Petri dishes were illuminated for 60 min or incubated in the dark. Illumination was provided by a set of four fluorescent tubes (Philips TL-D 18 W/840) at a distance of 60 cm, producing a light intensity of 7 W/m^2^. Mycelium samples were collected by filtration and immediately frozen at −80 °C for future use. For analysis of carotenoid production, standard 9-cm diameter Petri dishes with 25 mL solid medium were incubated at 30 °C for 7 days in the dark or under light as described above. Each Petri dish was inoculated with seven symmetrically distributed punctures with sterile sticks previously punctured on isolated fresh colonies. The mycelium samples were separated from the agar with a sterile blade and frozen for lyophilization. Carotenoid extractions were performed as described [20]. The statistical significance of the differences between the carotenoid values was assessed with the one sample *t* test, using Graphpad Prism version 8.0.2 for Windows (GraphPad Software, San Diego, CA, USA).

### 4.2. PCR Assays

Depending on the purpose of the experiment, different thermostable polymerases were used for PCR reactions: BIOTAQ^TM^ DNA polymerase (Bioline, Memphis, TN, USA) was used for PCR assays, Expand High Fidelity PCR System (Roche, Mannheim, Germany) for plasmid construction, and Velocity DNA polymerase (Bioline) for PCR products exceeding 5 kb. The reaction conditions were those indicated in manufacturer’s instructions. The reactions were carried out in total volumes of 25 μL with an amount of template DNA ranging from 10 to 100 ng for genomic DNA and from 1 to 10 ng for plasmid DNA. The primer sets used are described in Table 2.

### 4.3. Plasmid Constructions and Transformation

Plasmid pRS426-neocarP for the reintroduction of the *carP* sequence in *F. fujikuroi* was constructed by homologous recombination of three DNA segments, with overlapping end-sequences of at least 20 bases. One DNA segment was the 5.7-kb yeast vector pRS426 (Fungal Genetics Stock Center, [26]), which contains the *URA3* gene of *S. cerevisiae*, digested with *Xho*I and *Eco*RI at its multicloning site. The second was a 1980 bp DNA segment containing the *neoR* cassette obtained by PCR from plasmid pNTP2 with primer set 1 (PS1). pNTP2 is a 4.8-kb plasmid containing the neomycin phosphotransferase II gene (*nptII*) fused with cauliflower mosaic virus 35S promoter and terminator sequences. The third DNA segment contains the *carP* sequence, surrounded by 5′ and 3′ UTR regions to a final size of 2960 bp, obtained by PCR with PS2. Both primer sets had tails overlapping with the ends of *Xho*I/*Eco*RI digested pRS426 on one side, and with the end of each of the two other fragments on the other side. The three fragments were introduced by transformation into the *S. cerevisiae* strain FY834 (*MATα, ura3-52, leu2*Δ*1, trp1*Δ*63, his3*Δ*200, lys2*Δ*202*), where they recombined through the matching end-sequences to generate the 10.3 kb plasmid pRS426-neocarP. Protoplasts of strain SG268 were obtained and transformed with this plasmid as described [1]. For selection, geneticin (G418 disulfate salt; Formedium, Hunstanton, UK) was added to the medium at a final concentration of 150 mg/L.

### 4.4. Reintegration of Wild-Type carP in SF268

Protoplasts of SG268 were incubated with linear plasmid pRS246-carPneo and 32 transformants were obtained after selection on G418-supplemented medium. After three passes through uninucleate spores, the insertion of the native *carP* sequence in the candidate transformants was analyzed by PCR (Supplementary Figure S4) using different primer combinations (Table 2). In all transformants, a *carP* 1.2 kb band was obtained with PS3, indicating the presence of integrated plasmid containing the *carP* gene. Integration in the native *carP* locus (in situ integration) was only observed in transformants T3 and T18, as indicated by amplification with PS4 of a 2.2 kb region spanning from the start of *carP* to an external sequence in the *carS* promoter region. Southern blot hybridization of genomic DNA from the wild-type strain, SG268 Δ*carP* mutant, and four of the transformants (two with in situ integrations of *carP* and two with ectopic integrations), was carried out using as a probe a DNA segment next to *carP* (Supplementary Figure S4), included in the complementation plasmid. Genomic DNA samples were digested with restriction enzymes *Sph*I and *Xho*I. The results in the *Sph*I-digested samples showed a 10.3-kb band in the wild-type strain and a 10.5-kb band in SG268 and the negative transformants, while a 6-kb band was expected in the positive transformants. In samples digested with *Xho*I, the 4.9-kb band corresponding to the presence of the HygR cassette was found in the SG268 mutant and the transformants with ectopic integration of *carP*, while the 3.1-kb band expected for the *carP* sequence was found in the wild-type strain and in the transformants with in situ integration, confirming the results of the PCR tests and the hybridization of the samples digested with *Sph*I (Section 4.5). Thus, transformants T3 and T18 had recovered the deleted *carP* sequence at its native locus, while T2 and T21 had integrated it in other genomic locations.

### 4.5. Southern Blot

Samples of at least 4 μg of genomic DNA were digested with restriction enzymes, electrophoresed in a 0.7% agarose gel using a digoxigenin-labelled molecular ladder (DIG marker III o VII, Roche, Germany), and subjected to the Southern protocol using a positively charged nylon membrane (Hybond-N, Amersham, UK). The transfer was performed by capillarity as described [27]. The probe used in the Southern blot of the *F. fujikuroi* transformants was obtained by PCR with PS5 (Table 2). Probe was labelled with digoxigenin (PCR DIG Labeling Mix, Merck, Germany) according to manufacturer’s instructions. The membrane was preincubated with DIG Easy Hyb™ Granules (Roche) buffer solution for 1 h in a glass cylinder in a hybridization oven (HB-100 Hybridizer, UVP, CA, USA). The DIG-labelled probe was then added at 35 ng/mL to fresh buffer solution and incubated overnight. This was followed by two 5-min washes with 2x SSC 0.1% SDS and two 15-min washes with 2x SSC 0.1% SDS. Subsequently, the membrane was equilibrated with maleic buffer and incubated in blocking reagent solution (Roche) for one hour, and a stock of blocking solution with anti-DIG-alkaline phosphatase antibody (1:10,000) (Merck) was added for 30 min. Membrane was washed twice for 15 min with maleic buffer with 0.3% Tween-20, covered with detection buffer, and placed on an acetate. Then, CDP-Star^®^ ready to use (Roche) was added and the membrane was covered with another acetate. After a short incubation in the dark, the membrane’s signals were detected in the Odyssey Fc Imaging System (LI-COR, Lincoln, NE, USA).

### 4.6. Expression Studies

Total RNA was extracted with Trizol (Invitrogen, Paisley, UK) from 150–200 mg of ground mycelia samples, using the protocol described by the manufacturer. RNA concentrations were quantified with a Nanodrop ND-1000 spectrophotometer (Nanodrop Technologies, Wilmington, DE, USA). For qPCR measurements, 2.5 µg RNA sample was treated with DNAse I and retrotranscribed to cDNA with Transcriptor first-strand cDNA synthesis kit (Roche). Final cDNA concentrations were adjusted to 25 ng/μL. RT-qPCR analyses were performed in a LightCycler 480 real-time instrument (Roche) using LightCycler 480 SYBR Green I Master (Roche) following the manufacturer reaction protocol. The genes, primer sets, and amplicon sizes were *carB* (PS6, 68 bp), *carS* (PS7, 181 bp), and *carP* (PS8, 69 bp). Transcript levels of each gene were normalized against those of the β1-tubulin gene *FFUJ_04397* (PS9, 69 bp) and the glyceraldehyde-3-phosphate dehydrogenase (*gpdA*) gene *FFUJ_13490* (PS10, 94 bp). The data were relativized to the value of the wild-type strain grown in the dark, which was taken as 1.

### 4.7. RNA-seq Methodology

The experiments included three independent samples per strain and condition. Twenty μg of each RNA sample was treated with DNAse I in NucleoSpin RNA kit columns (Macherey-Nagel, Düren, Germany) following manufacturer’s instructions. The absorbance quality parameters (Ratio A260/A280 > 1.8 and A260/A230 > 1.5 and RIN > 8.5) were tested before processing the samples with the Agilent “Chip RNA Plant Nano” protocol by the company Life Sequencing (Valencia, Spain). Samples were sequenced on Illumina’s NextSeq platform in 75 bp single read mode. “Bcl2fastq2” version 2.19.1 provided by Illumina was used for the conversion of “bcl” files into “fastq” sequence files, a program that also removes the sequencing adapters. Quality values, number of readings, and total sequences, are shown in Supplementary Table S4.

### 4.8. Bioinformatic Analyses

Raw reads from all samples were trimmed, filtered, and quality controlled with AfterQC [28]. The sequences were mapped with Star [29]. The Integrative Genomics Viewer IGV application (IGV) version 2.8 was used for mapping visualization [30]. The annotation used was that from the EnsemblFungi database (https://fungi.ensembl.org, accessed on 20 August 2020) for *F. fujikuroi* IMI58289. The mapped sequences were analyzed using SeqMonk (version 1.45.4, https://github.com/s-andrews/SeqMonk, accessed on 25 July 2021). Quantification was performed using the RNA-seq quantitation pipeline with the improved mRNA annotation generated by Cuffmerge, merging transcripts, and counting reads over exons. Deseq2 tool [31], implemented in SeqMonk, which needs raw counts for quantitation, was used to compare among conditions. Differentially expressed genes were selected based on criteria combining a log2 fold change of 1 and an *FDR*-value of 0.05. Log2 RPM (reads per feature per million reads of library) were used for data visualization and intensity tests, which had a minimum *FDR*-value below 0.05 multiple testing correction applied with a sample size of 100 when constructing the control distributions. Venny 2.1 was used to draw Venn Diagrams (https://bioinfogp.cnb.csic.es/tools/venny/index.html, accessed on 10 April 2021). Gene expression levels were measured as TPM (transcripts per million) to represent the heatmaps. The heatmap figures were performed using the mean between the three samples in TPM for each strain and condition. The data were then log transformed, centered using the mean between them, and hierarchically clustered with Gene Cluster 3.0 [32]. The visualization was performed with Java TreeView3 [33].

FunCat GO enrichment results were obtained from the FungiFun database (https://elbe.hki-jena.de/fungifun/, accessed on 25 April 2021) using a significance level of 0.05 and testing for enrichment with FDR correction.

## Figures and Tables

**Table 1:** Numbers of genes whose expression changed more than two-fold above (upregulated) or below (downregulated) upon comparison of the indicated strains or culture conditions.

	Upregulated ^2^	% ^3^	Downregulated ^2^	% ^3^
Light ^1^ vs. dark in wild-type strain	970	6.4	860	5.7
Light ^1^ vs. dark in Δ*carP* mutant	270	1.8	192	1.3
Δ*carP* mutant vs. wild-type strain in the dark	71	0.4	40	0.2
Δ*carP* mutant vs. wild-type strain after light ^1^	148	1.0	405	2.7

^1^ 60 min illumination. ^2^ Upregulated (or downregulated) means increased (or decreased) transcript levels in illuminated compared to dark-grown samples, or in the Δ*carP* mutant compared to the wild-type strain. ^3^ Percentage referred to the total number (15,097) of genes annotated in the genome.

**Table 2:** Primer sets used in this work.

Primer Set		5′-3′ Sequence	Experimental Use
PS1	Forward	GTAACGCCAGGGTTTTCCCAGTCACACGGCTTGCCAACATGGTGGAGCACGACACTC	neoR segment pRS246neocarP
	Reverse	TGGATGACGCTTACTATAGTCTTGTCCCAACAAAAGCTGGAGCTCCACCGCGGTGGC	
PS2	Forward	GCCACCGCGGTGGAGCTCCAGCTTTTGTTGGGACAAGACTATAGTAAGCGTCATCCA	*carP* segment pRS246neocarP
	Reverse	GCGGATAACAATTTCACACAGGAAACAGCCAATCCGGGGACAATTCTAGAGGCACGCG	
PS3	Forward	CGTCGATGCGCCAGTTGATT	*carP* PCR
	Reverse	AGCAAGCGCCTAGTGGCC	
PS4	Forward	CGTCGATGCGCCAGTTGATT	*carP*-*carS* PCR
	Reverse	GTGTAGAGATTGGTGGGGGTT	
PS5	Forward	CCATTTCTGTTCCCTTCCCTG	*carP* probe for Southern
	Reverse	CCGTCATACACCAGAGAGAC	
PS6	Forward	TCGGTGTCGAGTACCGTCTCT	*carB* RT-qPCR
	Reverse	TGCCTTGCCGGTTGCTT	
PS7	Forward	GATACCCGGCGGAAAGGTTA	*carS* RT-qPCR
	Reverse	CTGACAGTCCATTTCAGCGC	
PS8	Forward	CCATTGAGCTGGGATGTGTTTT	*carP* RT-qPCR
	Reverse	TGCGCTGTGCTGTAAACCA	
PS9	Forward	CCGGTGCTGGAAACAACTG	Reference RT-qPCR *(*β_1_-tubulin)
	Reverse	CGAGGACCTGGTCGACAAGT	
PS10	Forward	GTGACCTCAAGGGCGTTCTG	Reference RT-qPCR *(gpdA*)
	Reverse	CGAAGATGGAGTTTGTGTT	

## Data Availability

The RNA-Seq data were deposited in NCBI’s Gene Expression Omnibus repository under GEO Series accession number GSE173210.

## Supplementary Materials

The following are available online at https://www.mdpi.com/article/10.3390/ncrna7030046/s1. Figure S1: Scatter plot representations on the effect of light and *carP* deletion on the *Fusarium fujikuroi* transcriptome. Figure S2: Funcat categories of the 680 genes downregulated by light in the wild-type strain. Figure S3: Funcat categories of genes differentially expressed in the Δ*carP* mutant. Figure S4: Molecular analysis of the reintegration of the *carP* sequence in SG268 by replacement with a G418^R^ cassette (*neoR* gene). Table S1: Data of the genes upregulated or downregulated by light or by Δ*carP* mutation. Table S2: Clusters of genes according to their induction or repression pattern in the wild-type strain and Δ*carP* mutant. Table S3: Funcat GO categories and genes in each category. Table S4: Data of the samples and sequences used in the RNA-seq analysis.
