# Supplementary material for: The carP lncRNA Is a carS-Related Regulatory Element with Broad Effects on the Fusarium fujikuroi Transcriptome
Source: Noncoding RNA. 2021 Aug 2;7(3):46. doi: 10.3390/ncrna7030046 (PMC8395912; doi:10.3390/ncrna7030046)
Supplement: Supplementary file 1 [file ncrna-07-00046-s001.zip › ncrna-1219813-Supplementary Materials - For XML.pdf]

non-coding RNA

MDPI

*Supplementary Materials*

# The *carP* lncRNA Is a *carS*-Related Regulatory Element with Broad Effects on the *Fusarium fujikuroi* Transcriptome

**Javier Pardo-Medina, Gabriel Gutiérrez, M. Carmen Limón and Javier Avalos ***

Departamento de Genética, Facultad de Biología, Universidad de Sevilla, 41012 Seville, Spain; jpardo6@us.es (J.P.-M.); ggpozo@us.es (G.G.); carmenlimon@us.es (M.C.L.)

* Correspondence: avalos@us.es; Tel.: +34-954-557-110

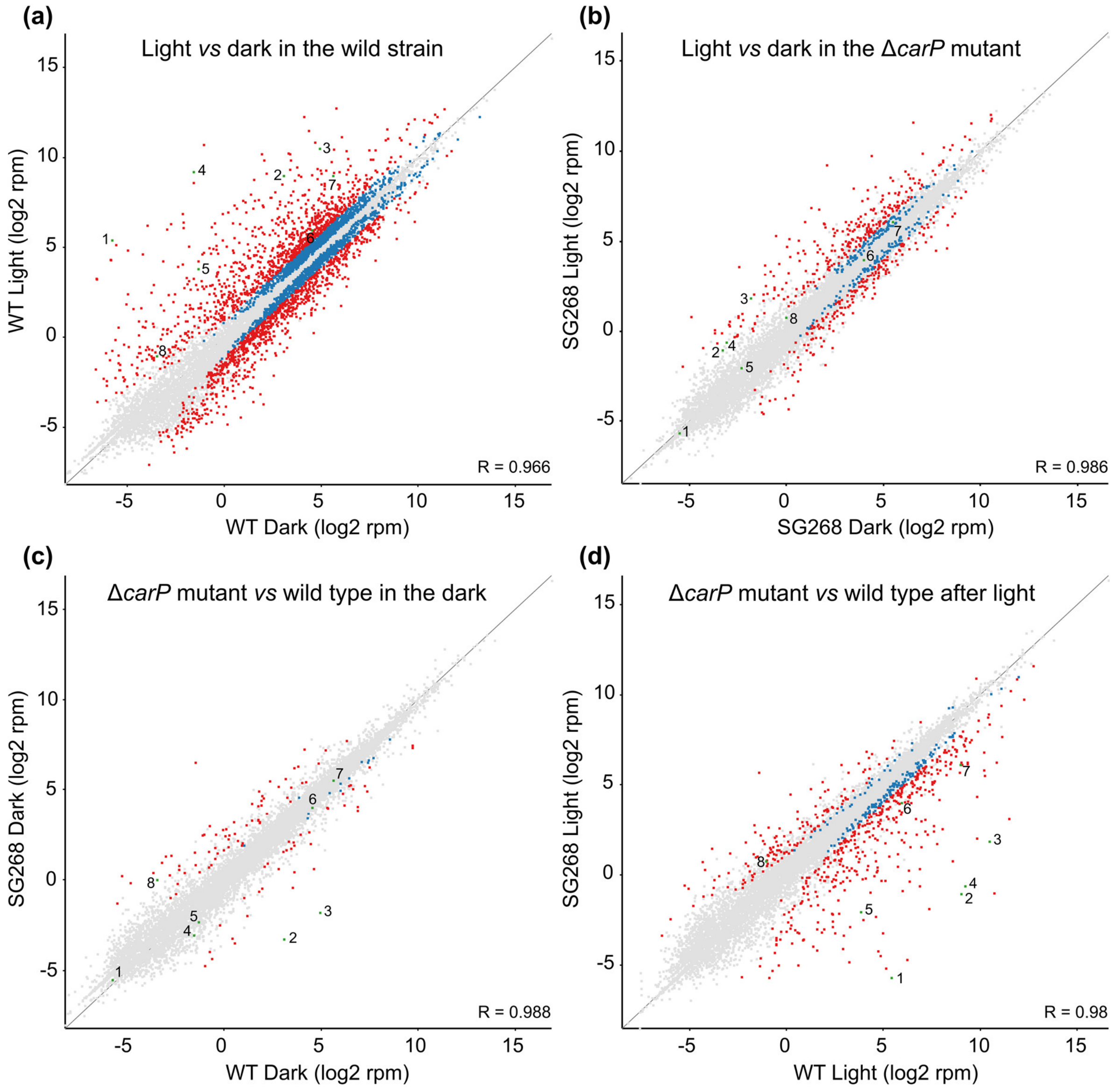


**Figure S1.** Scatter plot representations of the effect of light and *carP* deletion on the *F. fujikuroi* transcriptome. (**a**) Effect of light on the wild-type strain; (**b**) effect of light on the Δ*carP* mutant; (**c**) effect of the Δ*carP* mutation in the dark; (**d**) effect of the Δ*carP* mutation in light. Genes differentially expressed according to the Deseq analysis are indicated in blue. Genes exceeding the log2 values of + – 1 are indicated in red. Genes related to carotenoid metabolism are marked with green dots and a number. 1: *carX* (*FFUJ_11801*); 2: *carRA* (*FFUJ_11802*); 3: *carB* (*FFUJ_11803*); 4: *carO* (*FFUJ_11804*); 5: *carT* (*FFUJ_07962*); 6: *carD* (*FFUJ_07503*); 7: *ggs1* (*FFUJ_07352*); 8: *carS* (*FFUJ_08714*).

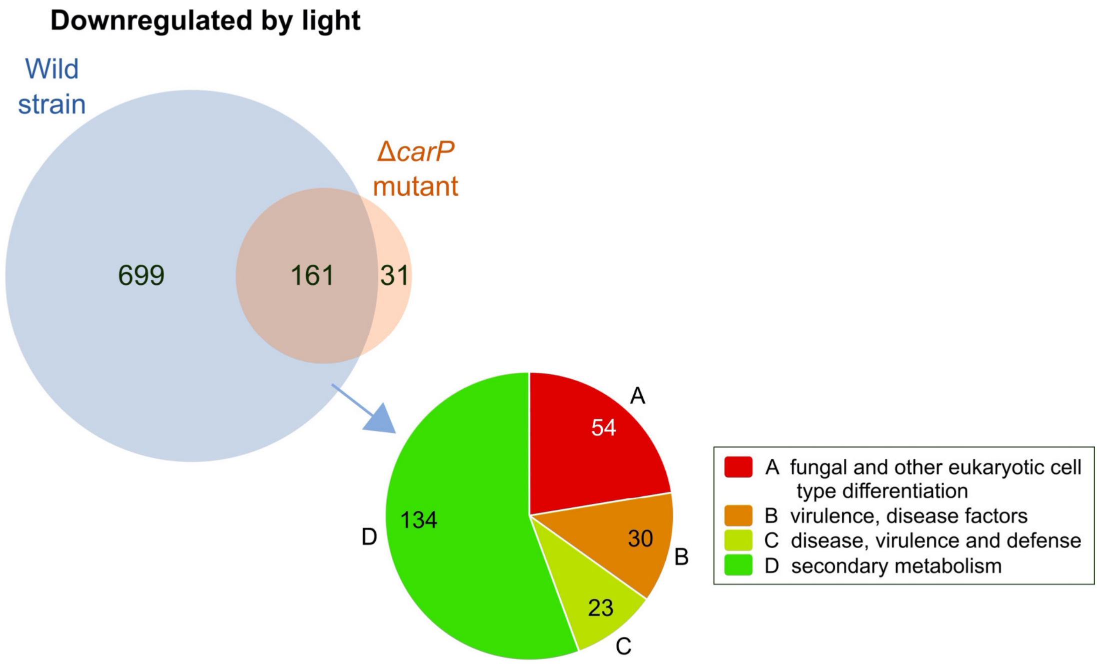


**Figure S2.** Funcat categories of the 680 genes downregulated by light in the wild strain. No significant Funcat categories were identified in the set of 192 transcripts from the Δ*carP* mutant. This is an extension of data displayed in Figure 2.

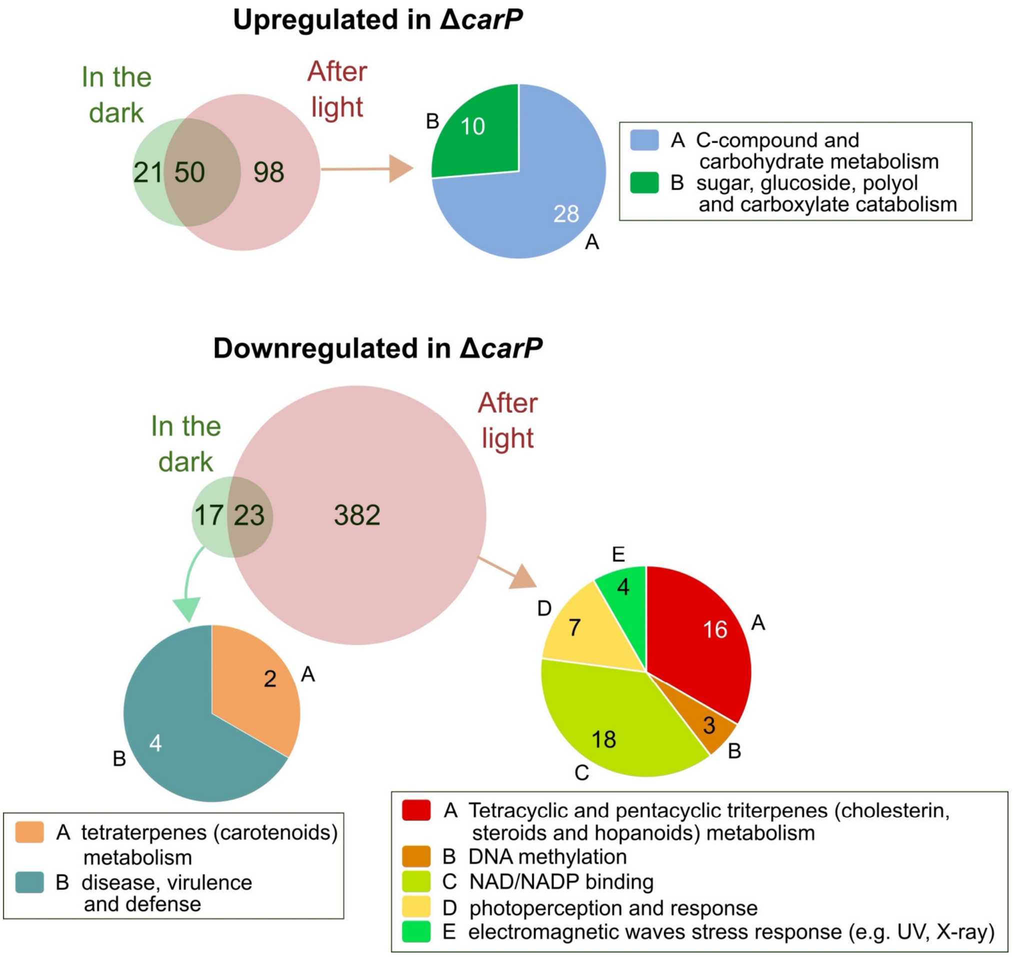


**Figure S3.** Funcat categories of genes differentially expressed in Δ*carP* mutant. Above, categories for the 148 genes upregulated after light. No significant categories were identified in the set of 71 genes upregulated in the dark. Below, categories for the 405 genes downregulated after light and the 40 genes downregulated in the dark. This is an extension of data displayed in Figure 3.

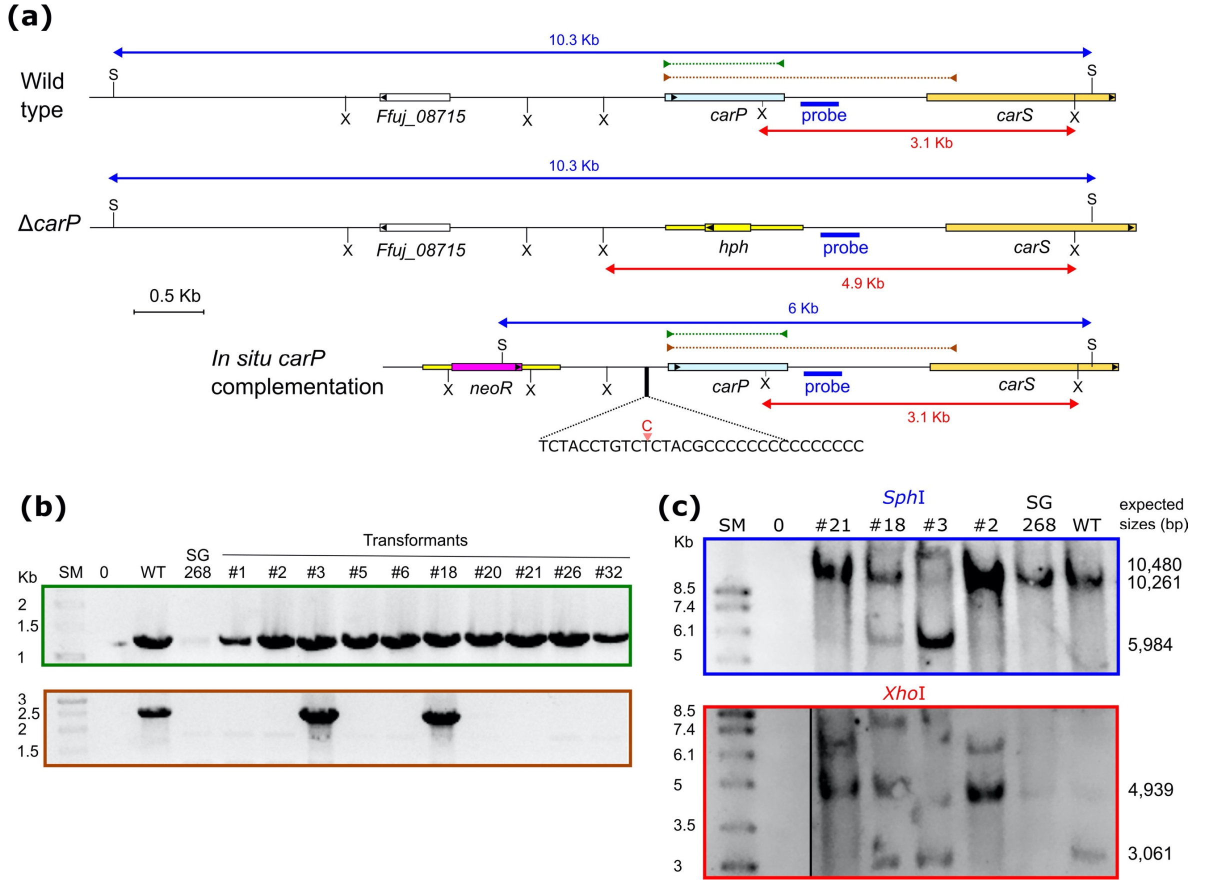


**Figure S4.** Molecular analysis of the reintegration of the *carP* sequence in SG268. (**a**) Genomic maps covering the area are shown for the wild strain, the Δ*carP* mutant SG268 and the *carP* reintegrated strains. The hygromycin resistance cassette (*hph* gene) was replaced with a G418$^R$ cassette (*neoR* gene). Deleted cytosine is featured in red in the *carP* reintegration map. (**b**) Gel electrophoresis of PCRs of candidate transformants to check the insertion of the cassette. DNA from the wild strain (WT) and SG268 Δ*carP* mutant, or lack of DNA (0), were used as controls. PCR amplification products are indicated on the upper map with colored arrowheads and dotted lines. Expected PCR sizes are 1245 bp (green), and 2194 bp (brown). (**c**) Southern blot of the wild strain (WT), SG268, and four candidate transformants. SM: Size markers. 0: DNA-free control. *Sph*I and *Xho*I restriction sites are indicated as S and X. The 412-bp hybridization probe (blue thick bar) and the expected hybridization fragments when DNA was digested with *Sph*I (blue arrowed line) and *Xho*I (red arrowed line), are indicated on the upper map.

Supplementary Table S1. Genes affected by light and carP mutation.

Supplementary Table S2. Clusters Heat map.

Supplementary Table S3. Funcat GO categories.

Supplementary Table S4. Data of RNAseq analysis.
